# Transmission Biology of Rice Stripe Mosaic Virus by an Efficient Insect Vector *Recilia dorsalis* (Hemiptera: Cicadellidae)

**DOI:** 10.3389/fmicb.2017.02457

**Published:** 2017-12-11

**Authors:** Xin Yang, Tong Zhang, Biao Chen, Guohui Zhou

**Affiliations:** Guangdong Province Key Laboratory of Microbial Signals and Disease Control, College of Agriculture, South China Agricultural University, Guangzhou, China

**Keywords:** rice stripe mosaic virus, cytorhabdovirus, *Recilia dorsalis*, transmission characteristics, rice disease

## Abstract

Rice stripe mosaic virus (RSMV) is a newly discovered species of cytorhabdovirus infecting rice plants that is transmitted by the leafhopper *Recilia dorsalis*. In this study, the transmission characteristics of RSMV by *R. dorsalis* were investigated. Under suitable growth conditions for *R. dorsalis*, the RSMV acquisition rate reached 71.9% in the second-generation population raised on RSMV-infected rice plants. The minimum acquisition and inoculation access periods of *R. dorsalis* were 3 and 30 min, respectively. The minimum and maximum latent transmission periods of RSMV in *R. dorsalis* were 6 and 18 d, respectively, and some *R. dorsalis* intermittently transmitted RSMV at 2–6 d intervals. Our findings revealed that the virus can replicate in the leafhopper body, but is likely not transovarially transmitted to offspring. These transmission characteristics will help guide the formulation of RSMV prevention and control strategies.

## Introduction

Rice (*Oryza sativa*) accounts for the majority of the world's cereal food production. Viral diseases are serious threats to rice production (Suzuki et al., 2015). Over the past few decades, yield loss has been caused by rice viruses in eastern and southeastern Asia, including repeated outbreaks of known and emerging new viruses. For example, *Rice tungro bacilliform virus* (RTBV, *Tungrovirus*) and *Rice tungro spherical virus* (RTSV, *Waikavirus*) in the Philippines and Indonesian (Hibino, 1996), *Rice stripe virus* (RSV, *Tenuivirus*) and *Rice black streaked dwarf virus* (RBSDV, *Fijivirus*) in China and Japan (Shikata and Kitagawa, 1977; Wang et al., 2009; Wu et al., 2009; Otuka et al., 2010; Otuka, 2013), *Rice gall dwarf virus* (RGDV, *Phytoreovirus*) in China and Southeast Asia (Omura et al., 1980; Zheng et al., 2015; Yang et al., 2017b) and *Rice ragged stunt virus* (RRSV, *Oryzavirus*) as well as a new species *Southern rice black-streaked dwarf virus* (SRBSDV, *Fijivirus*) in China and Vietnam (Anh et al., 2011; Otuka, 2013; Zhou et al., 2013; Wang et al., 2014), which all caused serious production losses. These viruses are transmitted by leafhopper or planthopper vectors, and controlling their transmission is the main way to prevent and control the spread of rice viruses.

Recently, we reported a new species of rice virus, rice stripe mosaic virus (RSMV), which occurs in southern China (Yang et al., 2017a). To date, this is the only reported cytorhabdovirus naturally infecting rice plants, and it causes slight dwarfing, yellow stripes, mosaic and twisted tips on leaves, unfilled grains and yield losses. RSMV is transmitted by the leafhopper *Recilia dorsalis*, but the characteristics of its transmission have not been sufficiently studied. To establish a disease control strategy based on vector control, the main RSMV transmission parameters of the leafhopper were investigated in this study.

## Materials and methods

### Rice plants, leafhoppers, and viral materials

The rice plants (*Oryza sativa* L. cv. “Nipponbare”) used in this study were grown as previously described (Yang et al., 2017a). The RSMV was obtained from infected rice field samples in Luoding, Guangdong Province, China during the 2016 growing season and confirmed by reverse transcription polymerase chain reaction (RT-PCR). Adult leafhoppers *R. dorsalis* and *Nephotettix cincticeps* were collected from a healthy field in Luoding and transferred to new cages with healthy rice plants at the tillering stage, when 3–4 branches have developed from the base of the seedling. To ensure that the insects were RSMV free, the second generation of the original insects that were confirmed RSMV negative by RT-PCR were reared on healthy rice seedlings with only three new leaves (3-leaf stage). From this population, one pair (male and female insects) were mated and reared on one healthy 3-leaf stage rice seedling that was replaced daily. Then, their offspring and the rice seedlings used to rear the insects were again tested by RT-PCR to confirm they were RSMV negative. The insects were propagated for over three generations before being used. The virus was transmitted by the leafhopper *R. dorsalis*, and maintained on rice plants growing in insect-proof greenhouses. The leafhoppers were maintained in insect-proof cages at 25°C and 80% relative humidity under a 16-h light/8-h dark photoperiod.

### RT-PCR detection of RSMV

Total RNAs of rice leaf tissues or leafhoppers were isolated using an RNeasy Plus Mini Kit (Qiagen, Gemany). RT-PCR amplification of the viral RNA was carried out using a One-Step RNA PCR kit (Takara, China). The virus-specific detection primers and PCR conditions were similar to those previously reported (Yang et al., 2017a).

### RSMV acquisition ability of *R. dorsalis*

*Recilia dorsalis* was allowed to feed on RSMV-infected rice plants at the tillering stage in the greenhouse for 1 month. Then, the second-generation's late-stage nymphs and adults were collected from the plants, and the presence of the virus in each individual insect was confirmed by RT-PCR.

### Acquisition access period of *R. dorsalis*

The RSMV-negative *R. dorsalis* 3rd–4th instar nymphs or adults were collected, starved for 4 h and then transferred to the RSMV-infected rice seedlings. The insects fed for 3, 5, 10, 30, 60, or 180 min and were then transferred to healthy 3-leaf stage rice seedlings in glass culture tubes (one plant and insect per tube) and maintained for 15 d for virus infection and propagation. The insects that were still alive after 15 d were collected and tested for the presence of the virus using RT-PCR. The viral acquisition access period of *R. dorsalis* was calculated from the RT-PCR results.

### Inoculation access period of *R. dorsalis*

The second-generation *R. dorsalis* 3rd–4th instar nymphs or adults propagated on RSMV-infected rice plants were collected, starved for 4 h, and then transferred to the healthy 3-leaf stage rice seedlings. The insects were allowed to feed on the seedlings for 3, 5, 10, 30, or 60 min, and were then individually tested using RT-PCR to determine whether they were RSMV positive. The seedlings inoculated with confirmed virus-positive leafhoppers were grown for 15 d. The inoculation access period of the leafhopper was determined from the results of the RT-PCR detection of the tested plants at 15 d after inoculation.

### Latent period of RSMV in *R. dorsalis*

To determine the latent period of RSMV in *R. dorsalis*, the method reported by Pu et al. (2012) was followed. Briefly, *R. dorsalis* nymphs at the 3rd–4th instar stage were fed on RSMV-infected rice plants for 12 h and transferred to healthy rice seedlings in glass culture tubes (one plant and insect per tube). The tested plants were replaced by healthy ones every 2 d until the insects died. Then, the tested plants were grown in an insect-proof greenhouse, and RT-PCR was conducted after 10 d. The latent period of RSMV in leafhopper was calculated from the RT-PCR results.

### Ability of *R. dorsalis* to transmit RSMV through eggs

To determine whether RSMV can be transovarially transmitted, the method reported by Chen et al. (2016) was followed. Briefly, the *R. dorsalis* adults propagated on RSMV-infected rice plants were collected, and 70 pairs (one male and one female) were transferred to healthy 3-leaf stage rice seedlings in glass culture tubes (one pair per tube). They were kept for 12 d and the seedlings were replaced daily. After the eggs were laid, the adults were individually tested by RT-PCR to determine whether they were RSMV positive. The eggs produced by females that were RSMV positive were transferred to healthy rice seedlings until they hatched, then the 3rd–4th instar nymphs were tested for RSMV.

### Electron microscopy

The *R. dorsalis* adults reared on RSMV-infected rice plants for more than 10 d were collected and their midgut tissues obtained. The tissues were excised on an ultra-microtome and fixed in 4% glutaraldehyde in cacodylate buffer (pH 7.4) at 4°C for 24 h, and then in 1% osmium tetroxide overnight. The fixed tissues were dehydrated through an alcohol series and embedded in Spurr's resin stained with 1% uranyl acetate and lead citrate (Li et al., 2015). Virion morphology and viroplasm distribution in the infected cells were examined under a transmission electron microscope (TECNAI G212, Holland).

### Determination of RSMV levels in leafhoppers by RT-qPCR

The RSMV-negative 3rd−4th instar *R. dorsalis* nymphs were fed on infected rice plants for 12 h and then transferred to healthy 3-leaf stage rice seedlings for 1, 5, or 10 d, with seedlings replaced daily. Total RNA (1 μg) from the insect was reverse-transcribed using the AMV RNA PCR Kit (Takara) in a 10-μL reaction mixture volume. The cDNA was diluted 10 times according to the manufacturer's protocol (PrimeScript® RT reagent Kit, Takara), and each qPCR reaction consisted of 2 μL cDNA, 0.5 μL of each primer (10 μM), 12.5 μL of SYBR®*Premix Ex Taq*™ II in a total volume of 20 μL. The PCR parameters consisted of one cycle at 95°C for 30 s, followed by 40 cycles of 95°C for 5 s and 60°C for 30 s. RT-qPCR reactions were carried out in a CFX96 Touch™ real-time PCR detection system (Bio-Rad, USA). The RSMV-specific primers N-F (5′-TAGAAGGGCGGCTACCTCA-3′) and N-R (5′-GACAGCCACATAGCGGAGAA-3′) were used for the viral content's relative quantification, and the *R. dorsalis*' *Actin* gene, with the primers Actin-F (5′-AGAAGTCCTACGAGTTGCCTGATG-3′) and Actin-R (5′-TTCATGATGGAGTTGTAGACGGTC-3′), was used as a reference (Chen et al., 2016). Over 30 leafhoppers were analyzed for each treatment, and three technical RT–qPCR replicates were performed for each biological replicate.

## Results

### RSMV acquisition ability of *R. dorsalis*

In the three experiments in which RSMV was detected in *R. dorsalis* adults collected from the second-generation populations raised on RSMV-infected rice plants, 66.7% (20/30), 80.0% (24/30), and 69.0% (20/29) of *R. dorsalis* adults were RSMV positive. The values were not significantly different. This indicated that *R. dorsalis* has a high frequency of virus acquisition from RSMV-infected rice plants after long-term feeding, and its viral mean acquisition rate can reach 71.9% (64/89).

### RSMV acquisition efficiency of *R. dorsalis*

The *R. dorsalis* nymphs and adults could become RSMV positive after feeding on the virus-infected rice plants for 3 min, with acquisition rates of 24.4 and 19.2%, respectively (Table 1). The acquisition rate gradually increased as the feeding time increased, and the rates in nymphs and adults increased to 66.7 and 58.9%, respectively, after 3 h of feeding (Table 1). Compared with adults, nymphs had higher acquisition rates (Table 1).

**Table 1:** The RSMV acquisition rate of *Recilia dorsalis* when feeding on RSMV-infected rice plants.

**Feeding time**	**Nymphs**			**Adults**		
	**No. of tested**	**No. of RSMV positive**	**Positive rate (%)**	**No. of tested**	**No. of RSMV positive**	**Positive rate (%)**
3 min	41	10	24.4	52	10	19.2
5 min	33	12	36.4	50	14	28.0
10 min	52	17	32.7	54	14	25.9
30 min	40	18	45.0	41	12	29.3
1 h	30	15	50.0	52	21	40.4
3 h	24	16	66.7	34	20	58.9

### RSMV inoculation efficiency of *R. dorsalis*

Neither the nymphs nor adults of virus-positive *R. dorsalis* could transmit RSMV to the tested plants within 10 min, but they successfully established infections in 15.0% and 5.0% of the tested plants, respectively, after 30 min of feeding (Table 2). The RSMV inoculation rate increased at 1 h of feeding, and the nymphs and adults could establish infections in 57.1 and 50.0% of the tested plants, respectively (Table 2).

**Table 2:** The transmission rates of RSMV by *Recilia dorsalis* individuals.

**Feeding time**	**Nymph**			**Adult**		
	**No. of tested**	**No. of infected**	**Transmission rate(%)**	**No. of tested**	**No. of infected**	**Transmission rate(%)**
3 min	29	0	0	26	0	0
5 min	20	0	0	22	0	0
10 min	20	0	0	20	0	0
30 min	20	3	15	20	1	5.0
1 h	28	16	57.1	24	12	50.0

### Latent period of RSMV in *R. dorsalis*

Using RT-PCR, 21 of the 32 tested leafhoppers were confirmed RSMV positive. The latent period of the virus in these 21 individuals was examined using viral transmission assays (Table 3). The minimum latent period of RSMV in *R. dorsalis* was 6 d (in insect no. 4) and the maximum period was 18 d (in insect no. 21). The latent period averaged 13 d (ranging from 6 to 18 d) under our experimental conditions. Some of the tested individuals (nos. 6, 7, 9, 13, 16, 19, and 21) could continuously transmit RSMV throughout their lives, but most of them intermittently transmitted the virus at intervals of 2–6 d. Three tested individuals (nos. 1–3) could not transmit the virus at any time during their lifespan.

**Table 3:** Latent period of RSMV in *Recilia dorsalis*.

**Vector No**.	**Days after virus acquisition**																		
	**2**	**4**	**6**	**8**	**10**	**12**	**14**	**16**	**18**	**20**	**22**	**24**	**26**	**28**	**30**	**32**	**34**	**36**	**38**
1					**D**														
2						**D**													
3							**D**												
4															**D**				
5																			**D**
6													**D**						
7															**D**				
8																**D**			
9																**D**			
10																	**D**		
11																	**D**		
12																			**D**
13														**D**					
14																		**D**	
15													**D**						
16														**D**					
17														**D**					
18														**D**					
19																**D**			
20																			**D**
21																		**D**	

*“**D**': tested insect died; “Light gray”: unable to transmit RSMV; “Dark gray”: able to transmit RSMV*.

### *R. dorsalis* could not transmit RSMV through eggs

Out of 70 pairs of leafhoppers that fed on RSMV-infected rice plant for the whole nymphal stage, 39 females were RSMV positive and 31 of their partner males were also virus positive. RSMV could not be detected in any of the 376 *R. dorsalis* individuals propagated from these 39 RSMV positive females, which indicated that RSMV was most likely not transmitted through the eggs of *R. dorsalis*.

### Propagation of RSMV in *R. dorsalis*

**Figure 1:**
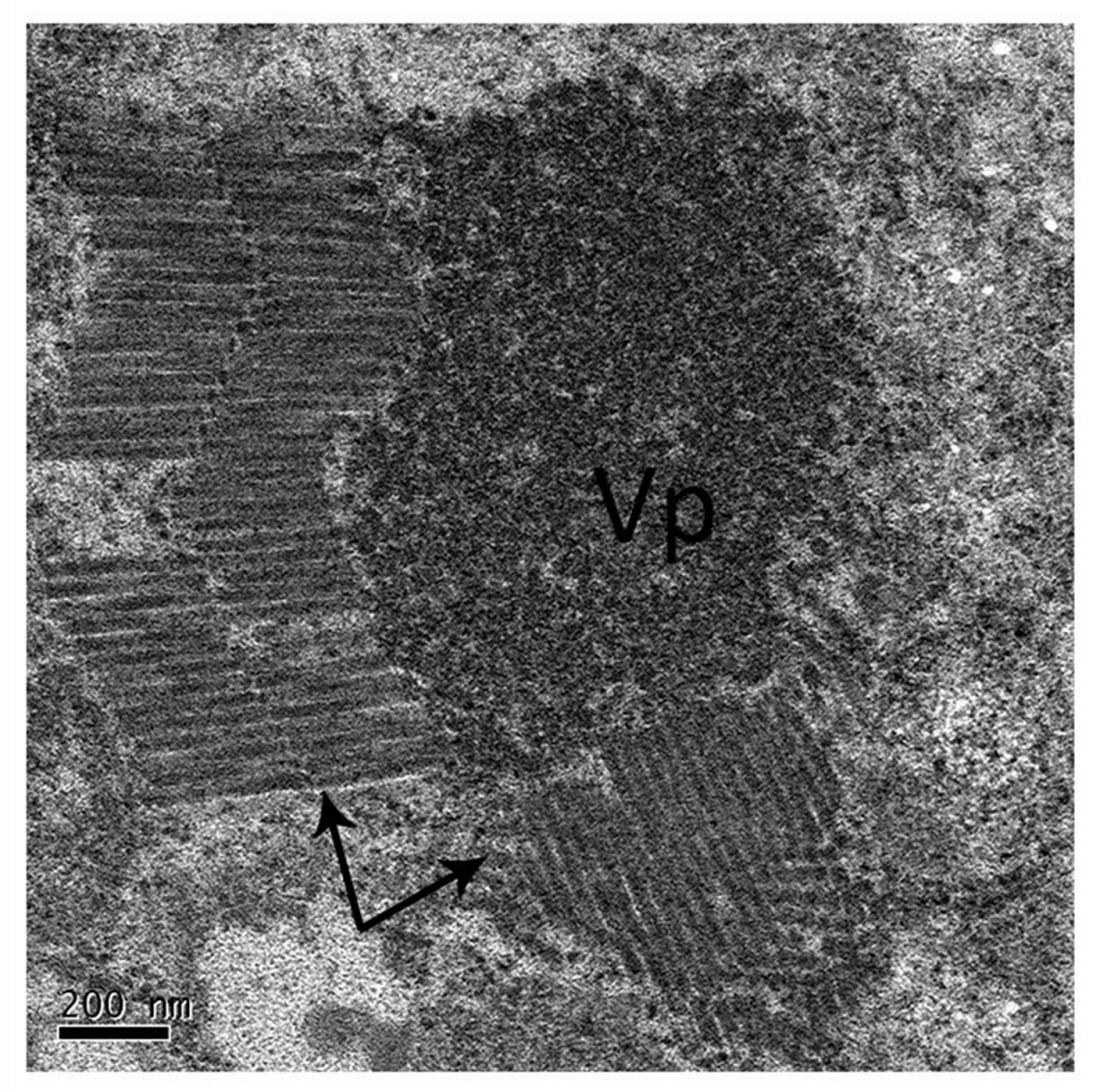
Electron microscopy of RSMV in the midgut of *R. dorsalis*. Midgut tissues of viruliferous *R. dorsalis* adults were dissected and negative-stained. RSMV virions were observed using a transmission electron microscope. “VP”: viroplasm; black arrows: RSMV virions.

The negative staining of midgut tissues from the RSMV-positive *R. dorsalis* revealed many bacilliform virions, having an average length of 325 nm and average width of 50 nm (*n* = 50), that were absent in the negative leafhoppers (Figure 1). This was consistent with our previous observations of RSMV virions in rice plants (Yang et al., 2017a). The bacilliform virions were mostly assembled into blocks and arranged in order. In particular, the virion blocks were adjacent to the electron-dense viral replication complex in the cytoplasm (Figure 1), which strongly suggests that the virions were formed by replication in *R. dorsalis*.

**Figure 2:**
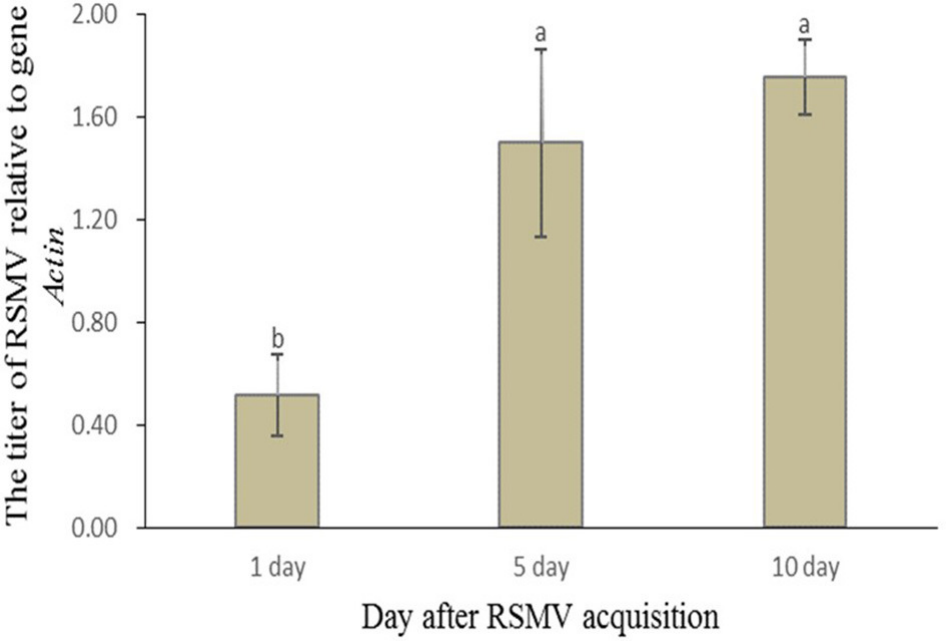
Relative RSMV titer in *R. dorsalis* after its acquisition from rice plants. Accumulation levels of the virus in *R. dorsalis* were normalized relative to the Actin gene at different time points as assessed by RT-qPCR. Each histogram bar represents the RSMV's relative mean titer from at least 30 individuals. Values followed by different letters represent significant differences based on a statistical analysis (SPSS 13.0) followed by Duncan's multiple range test at *P* < 0.05.

To further verify the propagation of RSMV in *R. dorsalis*, the viral titers in leafhoppers at different time points after acquisition were determined using RT-qPCR. The RSMV's mean titer in *R. dorsalis* showed an increasing trend after the leafhoppers left the virus-infected rice plants (Figure 2). The viral mean titer at d 5 (1.498) and 10 (1.756) were significantly higher compared with the mean titer at d 1 (0.517) after virus acquisition, but the mean titers at d 5 and 10 were not significantly different (Figure 2). These results indicated that RSMV propagated in the vector *R. dorsalis*.

## Discussion

Currently, the members of genus *Cytorhabdovirus* are mainly transmitted by planthoppers or aphids, except some viruses transmitted by unknown vectors (Jackson et al., 2005; Ammar et al., 2009). Interestingly, based on genome sequences, aphid-vectored viruses form a group, planthopper-vectored viruses form another group, and RSMV belongs to yet another subgroup (Yang et al., 2017a). Furthermore, nucleorhabdoviruses were similar to cytorhabdoviruses, which were also subgrouped based on their insect vectors (Yang et al., 2017a). In this study, RSMV is efficient transmitted by the leafhopper *R. dorsalis*, suggesting that this may be a useful feature for plant rhabdovirus classification.

Plant rhabdoviruses have a high degree of vector specificity and most are transmitted naturally by one or only a few closely related insect species (Jackson et al., 2005; Ammar et al., 2009). For example, *Barley yellow striate mosaic virus* (BYSMV) is mostly transmitted, with a high efficiency, by the small brown planthopper (*Laodelphax striatellus*), while another planthopper, *Javesella pellucida*, has a much lower transmission efficiency (Conti, 1980). *Wheat American striate mosaic virus* (WASMV) is transmitted by the leafhopper *Endria inimical*, while *Elymana virescens*, which is a close relative, only acts as a secondary transmission vector (Slykhuis, 1963; Sinsk, 1970). The two species of aphids *Hyperomyzus lactucae* and *Hyperomyzus carduellinus* can transmit *Lettuce necrotic yellows virus* (LNYV), but the former is the primary transmission vector (Randles, 1983). *R. dorsalis* is the efficient transmission vector of RSMV, and the second generation of *R. dorsalis* adults propagated on RSMV-infected rice plants have a very high RSMV-positive rate of over 70%. According to our field survey, *N. cincticeps*, another species of leafhopper, can also carry RSMV. However, the RSMV positive rate was significantly lower than that of *R. dorsalis*, and the virus transmission assay using *N. cincticeps* was also unsuccessful (unpublished data). Whether *N. cincticeps* is an additional vector of RSMV needs further investigation.

There are four basic types of insect vector–plant virus transmission relationships, non-persistent, semi-persistent, persistent-circulative and persistent-propagative (Whitfield et al., 2015; Dietzgen et al., 2016). To date, all of the known insect-vectored cytorhabdoviruses are transmitted in a persistent-propagative manner (Jackson et al., 2005; Hogenhout et al., 2008; Ammar et al., 2009). RSMV is also transmitted by *R. dorsalis* in a persistent-propagative manner, and the relative viral titer increases in the insect vector (Figure 2). Some other rice viruses, like RGDV and *Rice dwarf virus* (RDV, *Phytoreovirus*), can be transovarially transmitted to offspring of the common vector *R. dorsalis* (Zheng et al., 1997; Chen et al., 2016). However, RSMV was likely not transmitted to the offspring of viruliferous *R. dorsalis* adults under our experimental conditions, which is consistent with other plant rhabdoviruses transmitted by leafhoppers or planthoppers (Jackson et al., 2005; Ammar et al., 2009).

For plant viruses transmitted in the persistent-propagative manner, the virion is usually acquired by their insect vectors from the infected plants within a few hours. Both nymph and adult *R. dorsalis* acquired RSMV after 3 min of feeding on infected rice plants, and 3 h of feeding led to much higher acquisition rates (Table 1). This *R. dorsalis*' acquisition access period for RSMV is much shorter than those of many other insect transmitted viruses, like *Graminella sonora*-transmitted sorghum stunt mosaic rhabdovirus (SSMV), *L. striatellus*-transmitted BYSMV and *R. dorsalis*-transmitted RGDV (Conti, 1980; Morinaka et al., 1982; Creamer et al., 1997), which need 6, 5 and 8 h, respectively. The only know exception is WASMV. Its vector, *E. inimical*, needs only 30 s to acquire the virus (Slykhuis, 1963).

The latent period is required for the persistent-propagative transmission of viruses and is different lengths in different vector–virus combinations (Ammar et al., 2009). The latent period has important significance in viral epidemics and is also the basis of vector control-based disease prevention and control measures. The latent period of RSMV in *R. dorsalis* was 8–16 d (Table 3), which is longer than the 4 d of WASMV in *E. inimical* (Slykhuis, 1963) but similar to SSMV in *G. sonora, Rice yellow stunt virus* (RYSV) in *N. cincticeps* and SRBSDV in *Sogatella furcifera* (Chiu et al., 1968; Creamer et al., 1997; Pu et al., 2012). In addition, RSMV had a 2–6 d intermittent period during its transmission (Table 3), but the mechanism behind the interval is unclear. It may be caused by the insects' feeding behavior, which could involve frequent feeding on the plants during some stages but infrequent feeding during other stages. Another plausible explanation would be the induction of an immune response, such as autophagy or apoptosis, that lowers the transmission efficiency by reducing the amount of virus in the insects. Further studies on the mechanism of its intermittent transmission is required.

At least 30 min of feeding was needed for *R. dorsalis* to transmit RSMV to rice plants, and most needed 60 min of feeding (Table 2). Compared with other species of cytorhabdovirus, the inoculation access period of *R. dorsalis* transmitting RSMV was longer than that of *E. inimical* when transmitting WASMV, which takes only 15 min (Slykhuis, 1963), but was shorter than that of *G. sonora* transmitting SSMV, which takes 1 h (Creamer et al., 1997). Also, compared with other rice viruses, the inoculation access period of *R. dorsalis* transmitting RSMV was longer than that of *R. dorsalis* when transmitting RDV, which takes only 10 min (Shinkai, 1962), but was similar to that of *S. furcifera* transmitting SRBSDV, which takes 30 min (Pu et al., 2012).

Similar to most persistent-propagative transmission viruses (Creamer et al., 1997; Todd et al., 2010; Pu et al., 2012; Quito-Avila et al., 2012), the leafhopper nymphs seem to have higher RSMV acquisition and inoculation rates than adults, and the extended acquisition and inoculation times lead to higher rates (Tables 1, 2). If that is the case, then the spread of RSMV-associated diseases might occur mainly when nymphs are present in large numbers in the field, narrowing the window of time for viral spread to specific periods of the rice-growing season. These features should be taken into consideration when formulating disease prevention strategies.

The vector-mediated transmission of plant viruses is fascinating biologically and evolutionarily. However, there is little information available on genes or other factors involved in RSMV transmission. The rhabdoviruses encode glycoprotein, which is exposed to the surfaces of the viral particles and is essential for virus attachment to cell receptors (Jackson et al., 2005; Ammar et al., 2009; Dietzgen et al., 2016). Further studies clarifying the functions of RSMV glycoprotein in interactions with vector genes during viral infection may help facilitate investigations into the molecular and cellular bases of RSMV acquisition and transmission processes.

## Author contributions

GZ conceived and designed the experiments. XY performed the experiments and wrote the draft. TZ analyzed the data and revised the manuscript. BC prepared the materials. All authors read and approved the final manuscript.

### Conflict of interest statement

The authors declare that the research was conducted in the absence of any commercial or financial relationships that could be construed as a potential conflict of interest.
